# “AN INHUMANE LACK OF RESOURCES”: PERSPECTIVES ON PALLIATIVE AND HOSPICE CARE FOR UNHOUSED OLDER ADULTS

**DOI:** 10.1093/geroni/igad104.1192

**Published:** 2023-12-21

**Authors:** M Pilar Ingle, Asia Cutforth, Elise Matatall

**Affiliations:** University of Denver, Denver, Colorado, United States; University of Denver, Denver, Colorado, United States; Elevated Denver, Denver, Colorado, United States

## Abstract

Palliative and end-of-life (PEOL) care offer many benefits to those with life-limiting illness; however, older unhoused individuals face barriers in accessing these types of care. The purpose of this study was to explore healthcare and social service providers’ perspectives on approaches and challenges to addressing PEOL needs among unhoused individuals in Colorado. A descriptive, convergent mixed-methods design was used, including an online survey and semi-structured interviews. Participants were eligible if they worked in healthcare or social services in Colorado and provided direct care/services to adults. Guided by the Behavioral Model for Vulnerable Populations, the survey assessed satisfaction with care provided and identification of challenges in providing care along predisposing, enabling, and need contexts. Interviews focused on deeper accounts of provider experiences. Thematic analysis was used to evaluate qualitative data. Sixty-seven providers completed the survey, and 17 participated in interviews. Only 30% were satisfied with care provided to unhoused individuals with life-limiting illness within their organization. Lack of informal care support, mental illness, substance use, complex care needs, and lack of community resources were most frequently identified in the survey as challenges in providing care. Interviews highlighted person-centered and holistic approaches to care, as well as challenges of providing complex care in the context of an inhumane lack of resources. Tailored PEOL interventions, including street-based palliative care and social model hospice homes, were discussed as possible solutions. This study highlights humanizing approaches within structural challenges to providing PEOL care for unhoused individuals, and highlights potential opportunities to improve access to care.

